# Determinants of maternal health four weeks after delivery: cross-sectional findings from the KUNO-kids health study

**DOI:** 10.1186/s12889-021-11667-y

**Published:** 2021-09-15

**Authors:** Veronika Pinker, Susanne Brandstetter, Christina Tischer, Birgit Seelbach-Göbel, Michael Melter, Michael Kabesch, Christian Apfelbacher, The Kuno-kids study group, Andreas Ambrosch, Petra Arndt, Andrea Baessler, Mark Berneburg, Stephan Böse-O’Reilly, Romuald Brunner, Wolfgang Buchalla, Sara Fill Malfertheiner, André Franke, Sebastian Häusler, Iris Heid, Caroline Herr, Wolfgang Högler, Sebastian Kerzel, Michael Koller, Michael Leitzmann, David Rothfuß, Wolfgang Rösch, Bianca Schaub, Bernhard H. F. Weber, Stephan Weidinger, Sven Wellmann

**Affiliations:** 1 Institute of Social Medicine and Health Systems Research, Otto von Guericke University Magdeburg, Magdeburg, Germany; 2 Medical Sociology, Institute of Epidemiology and Preventive Medicine, University of Regensburg, Regensburg, Germany; 3 University Children’s Hospital Regensburg (KUNO) University of Regensburg, Regensburg, Germany; 4 Member of the Research and Development Campus Regensburg (WECARE), Hospital St. Hedwig of the Order of St. John, Regensburg, Germany; 5 Clinic of Obstetrics and Gynecology at St. Hedwig’s hospital, University of Regensburg, Regensburg, Germany

**Keywords:** Maternal health, Birth cohort study, Socioeconomic determinants, Self-rated health

## Abstract

**Background:**

The aim of this study was to examine the interaction of a multitude of socio-economic, lifestyle, environmental, psychosocial and birth related determinants and their effect on maternal health four weeks after delivery.

**Methods:**

We used data from a German birth cohort study, the KUNO-Kids health study. Social determinants, as well as the self-rated maternal health and the physical and mental health status of mothers (indicated by means of the SF-12-questionnaire) were assessed through standardized questionnaires and personal interviews right after delivery and four weeks later. Linear regression models were calculated to determine the relationship between influencing factors and health outcomes.

**Results:**

1428 women were included in the analysis. Maternal self-rated health showed significant positive associations with breastfeeding (B (regression coefficient) 2.67; 0.86–4.48 (95% Confidence interval)) and estimating one’s child as rather healthy (B 0.27; 0.19–0.34) and negative associations with social and emotional strains (B -3.50; -5.11- -1.88), obesity (B -2.56; -4.69- -0.42), having experienced a C-section (B -1.73; -3.23- -0.23), a positive history of somatic diseases (B -2.14; -3.53- -0.74), parental stress (B -0.39; -0.66- -0.11) and education of more than ten years (B -2.42; -3.95- -0.90).

Maternal physical health status showed significant negative associations with age (B -0.13; -0.25- -0.01), employment before maternity leave (B -1.90; -3.59- -0.21), social and emotional strains (B -1.50; -2.67- -0.34), parental stress (B -0.28; -0.45- -0.12), C-section (B -4.06; -5.12- -2.99), having the first child (B -2.03; -3.09- -0.97) and a history of somatic diseases (B -2.00; -2.99- -1.01).

Maternal mental health status showed significant positive associations with education of more than 10 years (B 2.27; 0.98–3.56) and a high level of social support (B 1.20; 0.06–2.34), while social and emotional strains (B -4.16; -5.48- -2.84) and parental stress (B -0.70; -0.92- -0.47) were negatively associated.

**Conclusions:**

We identified important protective factors for maternal health four weeks after delivery, such as a high level of social support. However, parental stress and social and emotional strains in particular seem to have a negative influence on maternal health. These findings have public health relevance.

**Supplementary Information:**

The online version contains supplementary material available at 10.1186/s12889-021-11667-y.

## Background

Several studies have investigated risk factors for poor psychological and physiologic health status of women during pregnancy and after delivery [1, 2]. Studies that focus on maternal health during pregnancy could show that there are significant negative associations between educational and occupation level and the prevalence of anemia in pregnant women [3], as well as between poverty and race and the occurrence of urinary tract infection, placenta disorders and preterm rupture of the membranes [4]. But not only the maternal health status during pregnancy but also the health status after delivery has been investigated earlier. Studies showed that higher maternal educational level and higher maternal occupational class as well as regular physical activity were associated with better maternal and better child health [5–8]. A study in Minnesota demonstrated that both physical and mental health problems 11 weeks postpartum (measured by the Short Form Questionnaire “SF-12” [9]) occurred more often in women with low coworker support and high job stress [10]. As a result, in particular the mental health status of mothers is considered at least as important as their physical health status. It could be shown that partnership and parity status, educational status, monthly income and residential property status were factors influencing depressive symptoms in mothers after delivery [11]. In addition, a study demonstrated that having children with chronic health conditions or developmental disabilities or activity limitations leads to a higher risk for adverse maternal mental health outcomes after delivery [12].

In general, previous studies focused mainly on one specific predictor for health outcomes whereas relatively little is known about the joint effects of numerous social determinants in relation to maternal health. In this study we use a comprehensive concept of health, including general, physical and mental health. A simultaneous consideration of socio-economic, environmental, lifestyle, psychosocial and birth related factors might help to identify women at risk for poor general, physical and mental health status four weeks after delivery. The current work extends prior work by examining the health status of mothers as well as a range of socioeconomic, environmental, lifestyle, psychosocial and birth and child related factors influencing maternal health outcomes using data from a prospective German birth cohort study.

## Methods

### Study design and study population

The study is based on data from the KUNO^1^-kids health study, a population-based, prospective birth cohort study conducted at the hospital St. Hedwig in Regensburg, Germany. St. Hedwig is located in Regensburg (153,000 inhabitants). The catchment area comprises the city of Regensburg as well as its mostly rural adjacent regions. Unemployment rates are low [13]. Being a tertiary perinatal center, the proportion of high-risk births is large. Rationale and design of the study have been reported in detail elsewhere [14]. For our study all women who came to St. Hedwig for the delivery of their baby (or babies) from June 2015 until June 2018 were asked to participate. Overall response was about 33% [14]. Participation was voluntary and written informed consent was obtained for each case. Participating women were asked to complete a standardized questionnaire in addition to a physical examination of the child. Researchers were trained to conduct interviews during a pilot study, using data not included in the final analysis. Exclusion criteria were inadequate German language skills (no basic German language skills for the comprehension of study procedures existent) and underage mother (< 18 years). The study was approved by the ethics board of the University of Regensburg (file number: 14–101-0347).

### Instruments

#### Assessment of social determinants

Maternal social determinants were assessed through a standardized questionnaire completed by mothers and, in addition, assessed by the KUNO-kids study team shortly after delivery. In addition, follow-up questionnaires were sent to the mothers four weeks after delivery. Socioeconomic determinants including age, family status, type of medical insurance, maternal education and employment before maternity leave, occupational group and maternal nationality were assessed within two days after delivery, as well as the level of social support and lifestyle factors, such as the level of physical activity, diet, height and weight before pregnancy, the number of doctors’ consultations during pregnancy and the history of psychiatric and somatic diseases. Further, birth related determinants, including birth mode, preterm delivery and season of birth were assessed right after delivery. Psychosocial factors such as the subjective position within society, the level of parental stress and social and emotional strains were assessed four weeks after delivery, as well as environmental and lifestyle factors, such as the housing situation, the drinking and smoking habits and secondhand smoke exposure. Child related determinants, including a visual analogue scale of the estimated child health “VAS child health”^2^ and the factor “breastfeeding” were also assessed four weeks after delivery.

We used the German version of the Mac-Arthur scale [15] to examine the mother’s subjective position within society. The level of social support (as experienced by the mother) was measured using the standardized short form social support questionnaire “F-SozU K-14”^3^ [16] [17]. It comprises the following domains: emotional and practical support, social integration, perceived social support and social strain. Every item can be answered on a scale of one (total rejection of the statement) to five (total agreement with the statement) out of which a mean score is calculated. To determine parental stress we used three scales of the EBI^4^ [18] a German version of the parenting stress index (PSI): parental competence, personal limitations and parent-child bond were each assessed with four items with a range of one to five points. Adding up the points for each domain results in a possible range of four to twenty points, with a higher score indicating a higher stress level. A total score was not calculated, as we only used three domains out of the original 12 domains.

For detailed information on all items, that have been used to assess social determinants **see** Table 1 and additional file 1**.**

**Table 1:** Assessment of socioeconomic, lifestyle, environmental, psychosocial and child and birth-related determinants

Social factors	Response options
*Socioeconomic factors (assessed within two days after delivery)*	
Age (years)	
Single mother	-yes (living alone, divorced, widowed, married but separated) -no (married, living with a partner)
Health insurance	- statutory - private - other (statutory and additional private, no insurance)
Maternal educational status	- < 10 years of education (Haupt/Volksschulabschluss) - 10 years of education (Realschulabschluss, Polytechnische Oberschule) - > 10 years of education (Abitur, Fachhochschulreife) - other (other type of graduation, not graduated yet, left school without graduation)
Employment status before maternity leave	- employed (fulltime employed, part time employed) - unemployed
Occupational group	- employee - worker - in training - self-employed - civil servant - graduate in liberal profession
Migration background	- yes - no
*Psychosocial factors (assessed within two days after delivery)*	
Social support index (F-Sozu)^1^	1–5
Psychosocial factors (assessed four weeks after delivery)	
Subjective position within society (Mac Arthur-Scale)	1–10
Experienced social and emotional strains	- yes - no
PSI^2^	
- Personal limitations	4–20
- Parental competence	4–20
- Parent-child bond	4–20
*Environmental and lifestyle factors (assessed four weeks after delivery)*	
Size of current residence (m^2^)	
Persons living in the household	
Humidity stains on the walls	- yes - no
Smoker inside the house	- yes - no
Drinking alcohol 1 year before pregnancy	- yes - no
Drinking alcohol during pregnancy	- yes - no
Drinking alcohol since delivery	- yes - no
Smoker lifetime (> 100 cigarettes)	- yes - no
Smoking during pregnancy	- yes - no
Smoking since delivery	- yes - no
*Lifestyle factors (assessed within two days after delivery)*	
BMI	- underweight (> 18.4 kg/m^2^) - normal (18.5–24.9 kg/m^2^) - overweight (20.1–29.9 kg/m^2^) - obese (> 30 kg/m^2^)
Physical activity	- regularly: more than 1 h per week - none or rather seldom: less than 1 h per week
Diet	- healthy: vegetables (not boiled) and/or fruits almost every day - rather unhealthy
*Health care utilization (assessed within two days after delivery)*	
Doctors’ consultations during pregnancy	
History of psychiatric diseases (depression, ADHD, anorexia, bulimia, panic attack)	- yes - no
History of somatic diseases (allergy, asthma, atopic dermatitis, Crohn’s disease, Colitis ulcerosa, psoriasis, psoriasis arthritis, rheumatic arthritis, other autoimmune diseases, diabetes, liver- or kidney diseases, thyroid diseases, cancer, thrombosis, arrythmia, heart attack, heart failure, hypertension, pyelonephritis, metabolic diseases, migraine, multiple sclerosis, peripheral nerve paralysis, epilepsy, meningitis, encephalitis)	- yes - no
Birth related factors (assessed within two days after delivery)	
Birth mode	
First child	- yes - no
Preterm delivery	- yes - no
Season of birth	- winter (September–February) - summer (March–August)
*Child related factors (assessed four weeks after delivery)*	
AS child health	1–100
Breastfeeding	- yes - no

^1^Only two missings acceptable, the mean value was calculated out of the remaining items.

^2^only one missing acceptable, the missing variable was replaced by the mean value of the remaining two items.

### Assessment of health outcome

#### Self-rated maternal health

Self-rated health was assessed four weeks after delivery on a visual analogue scale (VAS) ranging from 0 to 100 with 100 points indicating full health. The VAS is part of the EQ-5D^5^ [19].

#### Questionnaire assessed maternal health

Physical and mental health status of the mothers was determined by means of the “SF-12-questionnaire” four weeks after delivery [9]. This questionnaire is a validated tool that reflects the overall health-related quality of life [20]. The SF-12 asks how they would describe their general health status (excellent, very good, good, fair, poor). They were also asked if they experienced any new limitations with moderate daily activities, if they had difficulties accomplishing daily activities because of emotional or physical health problems, if they experienced any pain that would interfere with their normal work and how often they felt calm, energetic or disheartened. It was also asked how often physical health or emotional problems interfered with social activities during the last four weeks. SF-12 scores for both the physical and the mental component core range between 0 and 100 points with scores above and below 50 being above and below the average German population. A score above 50 represents a better physical or mental health than the population norm, respectively [21].

### Statistical analyses

The baseline characteristics of the study population were summarized descriptively. Linear regression was conducted to determine the relationship between health outcomes and potentially influencing factors. B-values and standardized ß-values were generated. Each variable with a *p*-value < 0.20 in univariable analysis was entered into a multivariable model to avoid missing variables that may show significant effects in multivariable analysis despite evident lack of associations in univariable analysis. For multivariable analysis the significance level was set to a p-value of < 0.05. All analyses were performed using SPSS 23 (IBM Corp., Armonk, NY, USA 2015). Mothers with missing data were excluded from the respective analyses.

## Results

Social and health characteristics of the sample of mothers are shown in Table 2. A total of 1428 of the 2788 women (51%) had completed the interview and the questionnaires right after delivery in addition to the questionnaire four weeks later and were therefore included in the analysis. Four weeks after delivery, the overall average self-rated health of mothers was 86.17 points (SD: 11.90), physical and mental component scores of SF-12 were 47.63 points (SD: 8.22) and 47.28 (SD: 10.64), respectively. The scores ranged from 19.02 to 68.53 (physical component scale) and from 7.87 to 65.84 (mental component scale).

**Table 2:** Characteristics of the study population

	Mothers (*n* = 1428)		
Characteristic	N	mean value / absolute number	[Minimum; Maximum] or %
*Socioeconomic factors*			
Age (years)	1414	34.45	19;49
Single mother	1404		
yes		25	1.8%
no		1379	98.2%
Health insurance	1402		
statutory		1170	83.5%
private		228	16.3%
other		4	0.3%
Educational status	1400		
None		2	0.1%
Low^1^		103	7.4%
Medium^2^		449	32.1%
High^3^		841	60.1%
Other		5	0.4%
Employment before maternity leave	1402		
Yes		1265	90.2%
No		137	9.8%
Occupational group	1259		
Employee		1001	79.5%
Worker		19	1.5%
In training		9	0.7%
Self-employed		42	3.3%
Civil servant		165	13.1%
Graduate in liberal profession		15	1.2%
Other		8	0.7%
Migration background	1428		
None		1272	90.4%
Yes		135	9.6%
*Psychosocial factors*			
Subjective position within society (Mac Arthur Scale)	1390	6.70	1.00;10.00
F-Sozu Score	1399	4.45	1.21;5.00
Social and emotional strain	1382		
Yes		369	26.7%
No		1013	73.3%
PSI			
Personal limitations	1420		
		10.02	4;20
Parental competence	1420		
		7.48	4;20
Parent-child bond	1413		
		8.41	4;20
*Domestic environment*			
Size of current residence per person living in the household (in m^2^)	1382	37.86	7.50;166.67
Humidity stains on the walls	1381		
Mold		186	13.5%
No		1195	86.5%
Smoking inside the house	1408		
Yes		7	0.5%
No		1401	99.5%
*Lifestyle factors*			
Drinking alcohol 1 year before pregnancy	1410		
Yes		933	66.2%
No		477	33.8%
Drinking alcohol during pregnancy	1418		
Yes		22	1.6%
No		1396	98.4%
Drinking alcohol since delivery	1414		
Yes		214	15.1%
No		1200	84.9%
Smoker lifetime (> 100 cigarettes)	1407		
Yes		601	42.7%
No		806	57.3%
Smoking during pregnancy	1384		
Yes		30	2.2%
No		1354	97.8%
Smoking since delivery	533		
Yes		38	7.13%
No		495	92.8%
BMI before pregnancy	1412		
underweight		31	2.2%
normal		857	60.7%
overweight		351	24.9%
obese		173	12.3%
Diet	1401		
Healthy		663	47.3%
Rather unhealthy		738	52.7%
Physical activity	1414		
None or rather seldom		636	45.0%
Regularly		778	55.0%
*Health care utilization*			
Doctors’ consultations during pregnancy	1422		
		4,66	0.86
History of psychiatric diseases	1245		
Yes		141	11.3%
No		1104	88.7%
History of somatic diseases	1400		
Yes		543	38.8%
No		857	61.2%
*Child and birth related factors*			
Birth mode	1428		
C-section		396	27.7%
Other		1032	72.3%
First child	1419		
Yes		821	57.9%
No		598	42.1%
Preterm delivery	1420		
Yes		85	6.0%
No		1335	94%
Season of birth	1428		
Winter		696	48.7%
Summer		732	51.3%
VAS child	1400	93.45	5;100
Breastfeeding	1414		
Yes		1156	81.8%
No		258	18.2%
*Outcomes*	N	mean value	[Minimum; Maximum]
Self-rated health mothers:	1355		
		86.17 (SD:11.90)	8;100
SF-12-Score^4^:	1345		
Mental component score (MCS)		47.28 (SD:10.64)	7.87;65.84
Physical component score (PCS)		47.63 (SD:8.22)	19.02;68.53

^1^Low: Haupt−/Volksschulabschluss in Germany.

^2^Medium: Realschulabschluss, Polytechnische Oberschule.

^3^High: Fachhochschulreife, Abitur

^4^No missings acceptable.

### Univariable analysis

Results of univariable analyses are presented in Tables 3 and 4**.**

**Table 3:** Associations between self-rated health and potential determinants in mothers: results of univariable linear regression analysis

Mothers (*n* = 1225)				
Self-rated health (0–100)				
Characteristic	B (CI 95%)	SE (B)	β	p
*Socioeconomic factors*				
Age (years)	0.06 (−0.09;0.21)	0.08	0.02	0.42
Single mother	0.28 (−4.76;5.31)	2.57	0.00	0.92
Health insurance	−0.27 (−2.01;1.48)	0.89	− 0.01	0.76
Educational status				
10 years	ref	ref	ref	
Less than 10 years	−0.37 (−3.00;2.26)	1.34	−0.01	0.78
More than 10 years	−1.34 (−2.74;0.05)	0.71	− 0.06	0.06
Employment before maternity leave	−1.08 (−3.28;1.12)	1.12	−0.03	0.34
*Psychosocial factors*				
Subjective position within society (Mac-Arthur Scale)	0.69 (0.18;1.19)	0.26	0.07	0.01
F-Sozu Score	2.54 (1.26;3.82)	0.65	0.11	< 0.01
PSI				
Personal limitation	−0.74 (− 0.89;-0.58)	0.08	− 0.25	< 0.01
Parental competence	−0.82 (− 0.99;-0.65)	0.09	− 0.25	< 0.01
Parent-child bond	− 0.64 (− 0.83;-0.45)	0.10	− 0.18	< 0.01
Social and emotional strains	−5.81 (−7.23;-4.40)	0.72	− 0.22	< 0.01
*Domestic environment*				
Size of current residence per person living in the house	− 0.00 (− 0.04;0.04)	0.02	− 0.00	0.91
Humidity stains (mold)	−0.87 (−2.75;1.02)	0.96	− 0.03	0.37
Smoking inside the house	4.14 (−5.43;13.70)	4.88	0.02	0.40
*Lifestyle factors*				
Drinking alcohol 1 year before pregnancy	− 0.48(− 1.83;0.88)	0.69	− 0.02	0.49
Drinking alcohol during pregnancy	1.84(−3.42;7.10)	2.68	0.02	0.49
Drinking alcohol since delivery	−0.73 (−2.51;1.06)	0.91	−0.02	0.42
Smoker Lifetime (> 100 cigarettes)	−1.08 (−2.38;0.21)	0.66	− 0.05	0.10
Smoking during pregnancy	−0.81 (−5.22;3.60)	2.25	−0.01	0.72
BMI (before pregnancy)				
Underweight	−1.55 (−6.00;2.88)	2.26	−0.02	0.49
Normal	ref	ref	ref	
Overweight	−0.35 (−1.86;1.17)	0.77	− 0.01	0.66
Obese	−2.04 (−4.03;-0.04)	1.02	−0.06	0.05
Healthy diet	0.35 (− 0.93;1.64)	0.66	0.02	0.59
Regular physical activity	0.57 (−0.71;1.86)	0.66	0.02	0.38
*Health care utilization*				
Doctors consultations during pregnancy	−0.13(− 0.24;-0.02)	0.06	− 0.07	0.01
History of psychiatric diseases	−2.75(−4.95;-0.55)	1.12	−0.07	0.01
History of somatic diseases	−2.51(−3.82;-1.20)	0.67	− 0.10	< 0.01
*Child and birth related factors*				
Birth mode (C-section)	−2.25(−3.65;-0.84)	0.72	−0.09	< 0.01
First child	−0.67(−1.97;0.62)	0.66	−0.03	0.31
Preterm delivery	0.60(−2.09;3.28)	1.37	0.01	0.66
Season of birth (winter)	−0.32(− 1.59;0.95)	0.65	− 0.01	0.62
VAS child	0.34(0.27;0.41)	0.04	0.26	< 0.01
Breastfeeding	2.07(0.42;3.73)	0.84	0.07	0.01

**Table 4:** Associations between SF-12-PCS and -MCS and potential determinants in mothers: results of univariable linear regression analysis

Mothers (n = 1225)								
	SF-12							
	Physical component scale				Mental component scale			
Characteristic	B (CI95%)	SE (B)	β	p	B (CI95%)	SE (B)	β	p
*Socioeconomic factors*								
Age (years)	−0.16	0.05	− 0.09	< 0.01	0.19	0.07	0.08	< 0.01
	(−0.26;-0.06)				(0.06;0.33)			
Single mother	−1.43	1.70	−0.02	0.40	−0.60	2.20	−0.01	0.79
	(−4.75;1.90)				(−4.91;3.72)			
Health insurance	−0.10	0.62	− 0.00	0.88	1.81	0.80	0.06	0.02
	(−1.31;1.12)				(0.24;3.38)			
Educational status								
10 years	ref	ref	ref		ref	ref	ref	
Less than 10 years	0.56	0.91	0.02	0.54	1.34	1.18	0.03	0.26
	(−2.28;-0.34)				(−0.98;3.65)			
More than 10 years	−1.31	0.50	−0.08	0.01	1.35	0.64	0.06	0.04
	(−2.28;-0.34)				(0.09;2.61)			
Employment before maternity leave	−1.32	0.76	−0.05	0.08	−1.56	0.98	−0.04	0.11
	(−2.81;0.18)				(−3.49;0.37)			
Migration background	−1.44	0.79	−0.05	0.07	−2.05	1.02	−0.06	0.05
	(−2.99;0.12)				(−4.06;-0.04)			
*Psychosocial factors*								
Subjective position within society (Mac-Arthur Scale)	0.17	0.18	0.03	0.35	0.85	0.23	0.10	< 0.01
	(−0.19;0.53)				(0.39;1.31)			
F-sozu score	0.51	0.45	0.03	0.26	3.85	0.577	0.18	< 0.01
	(−0.38;1.39)				(2.73;4.97)			
Social and emotional strains	−2.74	0.52	−0.15	< 0.01	−7.89	0.63	−0.33	< 0.01
	(−3.75;-1.73)				(−9.12;-6.66)			
PSI								
Personal limitation	−0.33	0.06	− 0.16	< 0.01	−1.27	0.07	− 0.47	< 0.01
	(−0.44;-0.22)				(− 1.39;-1.14)			
Parental competence	−0.21	0.06	−0.09	< 0.01	−1.45	0.07	−0.48	< 0.01
	(−0.33;-0.08)				(−1.59;-1.31)			
Parent-child bond	−0.14	0.07	−0.06	0.04	−1.17	0.08	−0.37	< 0.01
	(−0.27;− 0.01)				(−1.33;-1.01)			
*Domestic environment*								
Size of current residence per person living in the house	0.01	0.01	0.01	0.74	-0.01	0.02	− 0.02	0.78
	(− 0.02;0.03)				(−0.04;0.03)			
Humidity stains (mold)	−1.94	0.67	−0.08	< 0.01	− 0.53	0.87	− 0.02	0.55
	(−3.25;-0.64)				(−2.24;1.18)			
Smoking inside the house	3.89	3.11	0.03	0.21	−3.35	4.37	−0.02	0.44
	(2.21;9.98)				(−11.05;4.78)			
*Lifestyle factors*								
Drinking alcohol 1 year before pregnancy	− 0.83	0.48	−0.05	0.08	−0.52	0.62	−0.02	0.40
	(−1.77;0.10)				(−1.72;0.69)			
Drinking alcohol during pregnancy	−1.29	1.76	−0.02	0.47	−4.53	2.27	−0.05	0.05
	(−4.75;2.17)				(−9.00;-0.06)			
Drinking alcohol since delivery	−0.25	0.63	− 0.01	0.69	− 0.04	0.81	− 0.00	0.97
	(−1.48;0.98)				(− 1.62;1.55)			
Smoker lifetime (> 100 cigarettes)	0.55	0.46	0.03	0.23	−1.14	0.59	−0.05	0.05
	(− 0.34;1.45)				(−2.30;0.02)			
Smoking during pregnancy	1.16	1.55	0.02	0.46	−2.12	2.01	−0.03	0.29
	(−1.88;4.19)				(−6.05;1.82)			
BMI before pregnancy								
Underweight	−0.28	1.56	− 0.01	0.86	−1.30	2.01	−0.02	0.52
	(−3.33;2.78)				(−1.32;1.40)			
Normal	ref	ref	ref		ref	ref	ref	
Overweight	−0.40	0.54	−0.02	0.45	0.04	0.69	0.02	1.00
	(−1.45;0.65)				(−1.13;2.45)			
Obese	−0.60	0.71	−0.02	0.39	0.66	0.91	0.02	0.47
	(−1.99;0.78)				(−1.13;2.45)			
Healthy diet	0.19	0.45	0.01	0.67	1.07	0.59	0.05	0.07
	(−0.70;1.08)				(− 0.08;2.22)			
Regular physical activity	−0.33	0.45	−0.02	0.47	0.77	0.59	0.04	0.19
	(−1.22;0.57)				(−0.38;1.92)			
*Health care utilization*								
Doctors consultations during pregnancy	−0.11	0.04	−0.08	0.01	−0.02	0.05	−0.01	0.65
	−0.18;-0.03)				(−0.13;0.08)			
History of psychiatric diseases	−1.63	0.77	−0.06	0.03	−0.04	0.05	−0.86	0.03
	(−3.14;-0.13)				(−0.14;0.05)			
History of somatic diseases	−2.17	0.46	−0.13	< 0.01	−2.17	0.46	−0.13	< 0.01
	(−3.08;-1.26)				(−3.08;-1.26)			
*Child & birth related factors*								
Birth mode (C-section)	−4.44	0.49	−0.24	< 0.01	0.53	0.65	0.02	0.42
	(−5.39;-3.48)				(−0.75;1.80)			
First child	−1.40	0.46	−0.08	< 0.01	− 0.90	0.59	− 0.04	0.13
	(−2.30;-0.51)				(− 2.06;0.25)			
Preterm delivery	0.81	0.95	0.02	0.40	1.05	1.23	0.02	0.39
	(−1.06;2.68)				(−1.36;3.47)			
Season of birth (Winter)	0.01	0.45	0.00	0.99	−0.07	0.58	−0.00	0.90
	(−0.87;0.89)				(−1.21;1.06)			
VAS child	0.05	0.03	0.05	0.08	0.18	0.03	0.15	< 0.01
	(−0.01;0.10)				(0,11;0,24)			
Breastfeeding	−0.14	0.58	−0.01	0.81	0.17	0.75	0.01	0.83
	(−1.28;1.00)				(−1.31;1.64)			

#### Self-rated health

Breastfeeding, the mother’s subjective position within society, the level of social support (F-Sozu) and VAS child health showed significant positive associations with mothers’ self-rated health four weeks after delivery. Social and emotional strains, obesity, having experienced a C-section, a history of both psychiatric and somatic diseases, parental stress (PSI) and the number of doctors’ consultations during pregnancy were inversely associated.

#### SF-12-physical component scale (PCS)

The mother’s age, education of more than ten years, social and emotional strains, parental stress (PSI), humidity stains inside the house or flat, number of doctors’ consultations during pregnancy, having experienced a C-section, having the first child and a positive history of psychiatric and somatic diseases were negatively associated with the PCS-12 four weeks after delivery.

#### SF-12-mental component scale (MCS)

We found significant positive associations between mother’s age, health insurance, education more than ten years, mother’s subjective position within society, social support (F-Sozu), VAS child health and the MCS-12 four weeks after delivery: The older the mothers, the higher their subjective position within society, the higher their social support and the healthier mothers estimated their child, the better was their mental health status. Also having health insurance and having had an education of more than ten years showed better mental health status. Employment before maternity leave, migration background, social and emotional strains, parental stress (PSI), a history of somatic and psychiatric diseases and having the first child were negatively associated.

### Multivariable analysis

#### Self-rated health

Table 5 shows significant associations between determinants and the self-rated health of mothers four weeks after delivery. Breastfeeding and VAS child health remained as significant positive predictors in the multivariable analysis. Social and emotional strains, two of three domains of the PSI (personal limitations, parental competence), obesity, having experienced a C-section, and a positive history of somatic diseases were negatively associated with self-rated maternal health. In addition, education of more than ten years was also negatively associated with self-rated health of mothers.

**Table 5:** Associations between self-rated health and potential determinants in mothers: results of multivariable linear regression analysis

mothers (*n* = 947)				
	Self-rated health			
Independent variables	B (CI95%)	SE (B)	ß	p
Constant	64.74	5.40		< 0.01
	(54.15;75.34)			
Education less than 10 years	− 0.26	1.51	−0.01	0.87
	(−3.23;2.71)			
Education 10 years	*ref*	*ref*	*ref*	
Education more than 10 years	−2.42	0.78	−0.10	< 0.01
	(− 3.95;-0.90)			
Subjective position within society (Mac-Arthur scale)	0.50	0.30	0.05	0.10
	(0.09;1.09)			
F-sozu	0.46	0.71	0.02	0.52
	(−0.95;1.87)			
Smoker lifetime (> 100 cigarettes)	− 0.93	0.70	− 0.04	0.19
	(−2.30;0.44)			
Social and emotional strains	−3.50	0.82	−0.13	< 0.01
	(−5.11;-1.88)			
PSI-personal limitation	−0.31	0.12	−0.10	0.01
	(−0.54;-0.08)			
PSI-parental competence	−0.39	0.14	−0.12	0.01
	(−0.66;-0.11)			
PSI-parent-child bond	0.04	0.13	0.01	0.76
	(−0.22;0.30)			
BMI				
Underweight	−1.66	2.53	−0.02	0.51
	(−6.63;3.30)			
Normal	*ref*	*ref*	*ref*	
Overweight	0.24	0.81	0.01	0.77
	(− 1.35;1.83)			
Obese	−2.56	1.09	−0.07	0.02
	(−4.69;-0.42)			
Doctors consultations during pregnancy	−0.06	0.07	−0.03	0.33
	(−0.19;0.07)			
History of psychiatric diseases	0.09	1.12	0.00	0.93
	(−2.10;2.28)			
History of somatic diseases	−2.14	0.71	−0.09	< 0.01
	(−3.53;-0.74)			
Birth mode (C-section)	−1.73	0.76	−0.07	0.02
	(−3.23;-0.23)			
VAS child	0.27	0.04	0.19	< 0.01
	(0.19;0.34)			
Breastfeeding	2.67	0.92	0.09	< 0.01
	(0.86;4.48)			

R^2^ = 0.16

F = 8.12

#### SF-12-physical component scale (PCS)

Age, employment before maternity leave, social and emotional strains, one PSI domain (personal limitations), C-section, having the first child and a history of somatic diseases were negatively associated with the PCS-12. For detailed information see Table 6.

**Table 6:** Associations between SF-12-PCS and potential determinants in mothers: Results of multivariable linear regression analysis

mothers (*n* = 1044)				
	PCS-12^#^			
Independent variables	B (CI95%)	SE (B)	β	p
Constant	56.95	3.65		< 0.01
	(49.78;64.11)			
Age (years)	−0.13	0.06	− 0.07	0.04
	(− 0.25;-0.01)			
Education less than 10 years	−1.29	1.04	−0.04	0.21
	(−3.33;0.75)			
Education 10 years	*ref*	*ref*	*ref*	
Education more than 10 years	−0.84	0.55	− 0.05	0.13
	(−1.92;0.24)			
Employment before maternity leave	−1.90	0.86	−0.07	0.03
	(−3.59;-0.21)			
Migration background	−0.99	0.90	−0.03	0.27
	(−2.75;0.77)			
Humidity stains (mold)	−1.39	0.72	−0.06	0.05
	(−2.81;0.03)			
Drinking alcohol one year before pregnancy	−0.64	0.53	−0.04	0.23
	(−1.68;0.40)			
Social and emotional strains	−1.50	0.59	−0.08	0.01
	(−2.67;-0.34)			
PSI-personal limitation	−0.28	0.08	−0.13	< 0.01
	(−0.45;-0.12)			
PSI-parental competence	0.09	0.10	0.10	0.36
	(−0.11;0.29)			
PSI-parent-child bond	0.11	0.10	0.04	0.26
	(− 0.08;0.30)			
Doctors consultations during pregnancy	−0.08	0.05	−0.05	0.08
	(−0.18;0.01)			
History of psychiatric diseases	−0.56	0.80	−0.02	0.49
	(−2.13;1.01)			
History of somatic diseases	− 2.00	0.50	− 0.12	< 0.01
	(−2.99;-1.01)			
Birth mode (C-section)	−4.06	0.54	−0.54	< 0.01
	(−5.12;-2.99)			
First child	−2.03	0.54	−0.12	< 0.01
	(−3.09;-0.97)			
VAS child	0.04	0.03	0.05	0.13
	(−0.01;0.10)			

R^2^ = 0.12

F = 9.72

^#^physical component score of the short form 12 questionnaire

#### SF-12-mental component scale (MCS)

Education of more than 10 years and a solid social environment, indicated by a high score in the F-Sozu, showed significant positive associations with MCS-12. Social and emotional strains as well as two of three PSI domains (personal limitations and parental competence) showed significant negative associations with the MCS-12 (Table 7).

**Table 7:** Association between SF-12-MCS and potential determinants in mothers: Results of multivariable linear regression analysis

Mothers (*n* = 1031)				
	MCS-12^#^			
Independent variables	B (CI95%)	SE(B)	β	p
Constant	43.63	5.13		< 0.01
	(33.56;53.70)			
Age (years)	0.12	0.07	0.05	0.09
	(− 0.02;0.26)			
Health insurance	0.14	0.79	0.01	0.86
	(−1.40;1.68)			
Education less than 10 years	0.01	1.21	0.00	1.00
	(−2.36;2.37)			
Education 10 years	*ref*	ref	ref	
Education more than 10 years	2.27	0.66	0.10	< 0.01
	(0.98;3.56)			
Employment before maternity leave	−0.27	0.98	−0.01	0.78
	(−2.20;1.66)			
Migration background	−1.13	1.06	−0.03	0.28
	(−3.20;0.94)			
Subjective position within society (Mac-Arthur scale)	0.10	0.25	0.01	0.70
	(−0.39;0.59)			
F-sozu-Score	1.20	0.58	0.06	0.04
	(0.06;2.34)			
Drinking alcohol during pregnancy	−2.13	2.31	−0.02	0.36
	(−6.68;2.41)			
Smoker lifetime (> 100 cigarettes)	−0.36	0.57	−0.02	0.53
	(−1.47;0.75)			
Social and emotional strains	−4.16	0.67	−0.17	< 0.01
	(−5.48;-2.84)			
PSI-personal limitation	−0.68	0.10	−0.25	< 0.01
	(−0.87;-0.49)			
PSI-parental competence	−0.70	0.11	−0.23	< 0.01
	(−0.92;-0.47)			
PSI-parent-child bond	−0.17	0.11	−0.05	0.13
	(−0.38;0.05)			
Healthy diet	0.49	0.56	0.02	0.39
	(−0.62;1.59)			
Regular physical activity	0.03	0.58	0.00	0.95
	(−1.10;1.17)			
History of psychiatric diseases	−1.19	0.89	−0.04	0.18
	(−2.95;0.56)			
History of somatic diseases	0.35	0.57	0.02	0.54
	(−0.77;1.47)			
First Child	0.64	0.63	0.03	0.31
	(−0.59;1.88)			
VAS child	0.07	0.03	0.06	0.03
	(0.01;0.13)			

R^2^ = 0.32

F = 25.74

^#^mental component score of the short form 12 questionnaire

## Discussion

### Summary of main findings

The aim of our study was to provide a contribution to the literature on how a multitude of socio-economic, lifestyle, environmental, psychosocial and birth related determinants effects maternal health. We identified risk factors for maternal health outcomes four weeks after delivery, especially social and emotional strains, parental stress and education of more than 10 years. We could also determine positive factors for maternal health, such as a high subjective position within society or a high score in the VAS child.

### Comparison of findings with previous studies

Some of our findings are consistent with earlier studies, emphasizing the importance of a stable social environment for mothers’ health [1, 6]. Dennis et al. identified a lack of social support as a risk factor for mothers’ self-rated health. Also having had a C-section (or forceps or vacuum delivery), no or little physical activity and low income were negatively associated [1]. Some of our findings on the other hand seem to be surprising in the context of prior work. Education more than ten years appears to have a significant negative influence on one’s self-rated health. Former studies show that the self-rated health varies with perception of what constitutes good health and with expectations regarding one’s own health [22]. It is assumed that more highly educated individuals are better informed of treatment options and are less tolerant of a given health condition [23]. A study in Germany in 2010 showed that the higher the educational level of women, the higher the number of doctor’s consultations [24]. An explanation for this relation might be that the more knowledge mothers have, the more they tend to be worried about any complications and therefore report a lower self-rated health. Our finding warrants further investigation though.

We took the physical and mental component scale of the SF-12-questionnaire to picture the physical and mental health status of the mothers. Previous studies using the SF-12 found that job stress, amongst others, was a negative predictor for mental health status of mothers [10]. Interestingly, we found that employment before maternity leave was associated with a lower physical health status in mothers. This finding might inform a reconsideration of maternity protection in Germany.

### Strengths and limitations

Our work adds valuable new findings to already existing studies and knowledge about maternal health. First, we identified several potential risk factors for maternal health that have not been the focus of previous research, including a high maternal education or employment before maternity leave. To the best of our knowledge it is one of the first studies investigating the simultaneous influence of a multitude of factors, not focusing on only specific factors. It is important to understand how socioeconomic, environmental, lifestyle, psychosocial and birth related factors together influence maternal health outcomes. Health is a complex multidimensional construct. In our study we used a comprehensive approach to describe maternal health four weeks after delivery including the self-rated health of mothers on a visual analogue scale and the physical and mental component scales of the SF-12, a well-established and validated tool to measure health-related quality of life [9].

Previous studies tend to dichotomize self-rated health into two groups of poor/fair self-rated health and good/excellent self-rated health or use other items to describe it, like the Personal Health Scale (PHS) or the General Health Questionnaire (GHQ) [1, 25]. The visual analogue scale we used with a response range from 0 to 100 offers very fine nuances and prevents respondents of being bound to predetermined categories [26].

Some limitations of this study must be considered.

All of our data are self-reported and therefore subject to recall bias. To overcome this limitation, mothers were interviewed shortly after delivery and again after four weeks in order to potentially improve the mothers’ recall of events. All members of the study team were trained to conduct the interview in a professional and sensitive way. Still information may have been misreported due to social desirability bias [27].

The response rate in our study is 33% (investigated in detail during a 2.5 months’ time period) [14]. This seems low, compared to other earlier German birth cohort studies, such as the ULM-Spatz birth cohort study with a response rate of 48% or the GINIplus birth cohort study with a response rate of 55% [28]. Still, response rates between studies vary. Other birth cohort studies in Europe, such as the Danish national birth cohort study have achieved a similar response rate of 35% [29] and also data from the currently ongoing German National Cohort NAKO suggests that participation rates in population based studies are low [30]. In the KUNO-kids study potential differences between respondents and non-respondents have been investigated. The most frequent reasons for non-participation were insufficient German language skills, mothers’ perception that the study procedures are associated with too much effort and lack of interest [14]. For the analysis of determinants of maternal health we investigated differences between the initial sample of mothers who provided written informed consent and participated in the baseline interview and the final study population (who also returned the four-weeks-questionnaire). The rate of single mothers and mothers with migration background in the final study sample is lower compared to the original sample. Educational status and rate of employment before maternity leave in the final sample on the other hand are higher compared to the original study population (see additional file 2). Compared to data from registration offices our final study population has a lower rate of single mothers and mothers with migration background [13]. However, educational level in our study is similar to the educational level of women aged between 25 and 35 years in Germany [31] and child and birth related characteristics in our study reflect the average perinatal statistics in Bavaria with similar ratios of C-section or preterm delivery [32]. In summary, we cannot deny the presence of selection bias in our study. In the context of prior birth cohort studies this observation does not seem too surprising [33] as people from a higher social class tend to participate in health studies more often than people from a lower social class.

## Conclusion

We noticed that some risk factors for maternal health become more and more common, such as a high educational level for women, employment before maternity leave and high-aged primipara. As it could be shown in our study, these factors are associated with lower maternal self-rated health and a lower physical health status four weeks after delivery which underlines the importance of reinvestigating these relations. It appears interesting that we found an association between higher education and a lower self-rated health. This is against all previous findings and should definitively lead to more studies examining this relation.

By investigating several factors and their influence on maternal health the importance of mothers’ social support became clear. Social and emotional strains as well as parental stress seem to be negatively associated with both the self-rated health and the physical and mental health status of mothers. In this context the enforcement of social support networks, as well as stable family structures seem important. Concretely, it could reduce maternal stress and strains to establish family-friendly work concepts. Particularly in rural areas there is the need to extend social support offers, such as educational counseling or cry baby clinics. This could improve maternal health outcomes in the future.

## Supplementary Information

**Additional file 1.** Items, that have been developed for the KUNO-kids study

**Additional file 2.** Characteristics of the study population compared to the initial sample (mothers who participated only in the baseline interview)

## Data Availability

The datasets used and/or analyzed during the current study are available from the corresponding author on reasonable request.

## Abbreviations

EBI: Elterliches Belastungsinventar = German version of the Parenting Stress Index
EQ-5D: a standardized health-related quality of life questionnaire developed by the EuroQol Group
F-Sozu: Fragebogen zur sozialen Unterstützung = German social support questionnaire
K-14: Kurzform 14 = questionnaire with 14 questions
KUNO: Kinder Uniklinik Ostbayern
PSI: Parenting Stress Index
SF-12: Short form questionnaire
MCS: mental component scale
PCS: physical component scale
VAS: visual analogue scale

## Footnotes

1 KUNO = Kinder-Uniklinik Ostbayern = children’s university hospital in East Bavaria

2 VAS child health: visual analogue scale of the estimated child health

3 F-SozU K-14: Fragebogen zur sozialen Unterstützung (Kurzform 14) = German social support questionnaire with 14 questions

4 EBI: Elterliches Belastungsinventar = German version of the Parenting Stress Index

5 EQ-5D: a standardized health-related quality of life questionnaire developed by the EuroQol Group
