# Supplementary material for: Determinants of maternal health four weeks after delivery: cross-sectional findings from the KUNO-kids health study
Source: BMC Public Health. 2021 Sep 15;21:1676. doi: 10.1186/s12889-021-11667-y (PMC8442319; doi:10.1186/s12889-021-11667-y)
Supplement: Supplementary file 1 — Additional file 1. Items, that have been developed for the KUNO-kids study [file 12889_2021_11667_MOESM1_ESM.docx]

**Additional file 1:**

**List of items, developed for the KUNO-Kids study:**

- Wie alt sind Sie? (How old are you?)
- Sind Sie alleinerziehend? (Are you a single parent?)
  - Yes
  - No
- Wie sind Sie krankenversichert? (Which health insurance do you have?)
  - Statutory
  - Private
  - Other (statutory and additional private, no insurance)
- Welchen höchsten allgemeinbildenden Schulabschluss haben Sie? (Which is your highest level school leaving certificate?)
  - <10 years of education (Haupt-, Volksschulabschluss)
  - 10 years of education (Realschulabschluss, Polytechnische Oberschule)
  - >10years of education (Abitur Fachhochschulreife)
  - Other (other type of graduation, not graduated yet, left school without graduation)
- Waren Sie vor Eintritt in den Mutterschutz erwerbstätig, d.h. gingen Sie einer bezahlten Tätigkeit nach? (Have you had an occupation before maternity leave?)
  - Employed (fulltime employed, part time employed)
  - unemployed
- Zu welcher Berufsgruppe gehörten Sie zuletzt? (Which occupational group did you belong to last?)
  - Employee
  - Worker
  - In training
  - Self-employed
  - Civil servant
  - Graduate in liberal profession
- In welchem Land sind Sie geboren? (What is your birth country?)
- Haben sich in den letzten 4 Wochen besondere Belastungen für Sie ergeben? (Have you experienced any social or emotional strains during the last 4 weeks?)
  - Yes
  - no
- Wie groß ist Ihre Wohnung/Ihr Haus ungefähr? (How big is your apartment or house?)
- Wie viele Personen leben in Ihrem Haushalt? (How many persons, including the newborn, are currently living in your apartment/house?)
- Gab es in Ihrer Wohnung irgendwelche Feuchtigkeitsflecken oder Schimmel an Wänden oder Decken? (Have there been any humidity stains on the walls of your apartment/house?)
  - Yes
  - no
- Wird seit der Geburt Ihres Kindes in Ihrer Wohnung geraucht? (Is anybody smoking inside your apartment/house since the delivery of your child?)
  - Yes
  - No
- Haben Sie im Jahr vor dieser Schwangerschaft gelegentlich oder regelmäßig Alkohol getrunken? (Have you been drinking alcohol before the pregnancy?)
  - Yes
  - No
- Haben Sie während dieser Schwangerschaft gelegentlich oder regelmäßig Alkohol getrunken? (Have you been drinking alcohol during the pregnancy?)
  - Yes
  - No
- Haben Sie seit der Geburt gelegentlich oder regelmäßig Alkohol getrunken? (Have you been drinking alcohol since the delivery of your baby?)
  - Yes
  - No
- Haben Sie bisher in Ihrem Leben mehr als 100 Zigaretten geraucht? (Have you been smoking more than 100 cigarettes altogether in your life?)
  - Yes
  - No
- Haben Sie im Jahr vor dieser Schwangerschaft geraucht? (Have you been smoking in the year before the pregnancy?)
  - Yes
  - No
- Haben Sie während dieser Schwangerschaft geraucht? (Have you been smoking during pregnancy?)
  - Yes
  - No
- Haben Sie seit der Geburt Ihres Kindes regelmäßig Zigaretten? (Have you been smoking cigarettes regularly since the delivery of your baby?)
  - Yes
  - No
- Wie groß sind Sie? (What’s your height?)
- Wie schwer sind Sie? (What’s your weight?)
- Wie oft haben Sie während der jetzigen Schwangerschaft Sport getrieben? (How often did you exercise during pregnancy?)
  - Never
  - Less than one hour per week
  - Regularly 1-2 hours per week
  - Regularly more than 2 hours per week
- Welche der folgenden Nahrungsmittel nahmen Sie in der Schwangerschaft zu sich? (Which food products did you consume during pregnancy?)
  - Meat (once a month, several times a month, once a week, almost daily, never)
  - Sausages (once a month, several times a month, once a week, almost daily, never)
  - Poultry (once a month, several times a month, once a week, almost daily, never)
  - Fish (once a month, several times a month, once a week, almost daily, never)
  - Potatoes (once a month, several times a month, once a week, almost daily, never)
  - Pasta (once a month, several times a month, once a week, almost daily, never)
  - Rice (once a month, several times a month, once a week, almost daily, never)
  - Salad or not boiled vegetables (once a month, several times a month, once a week, almost daily, never)
  - Fruits (once a month, several times a month, once a week, almost daily, never)
  - Bread (once a month, several times a month, once a week, almost daily, never)
  - Crispbread (once a month, several times a month, once a week, almost daily, never)
  - Cereals (once a month, several times a month, once a week, almost daily, never)
  - Salty snacks (once a month, several times a month, once a week, almost daily, never)
  - Chocolate (once a month, several times a month, once a week, almost daily, never)
  - Cake (once a month, several times a month, once a week, almost daily, never)
  - other sweets (once a month, several times a month, once a week, almost daily, never)
  - Yoghurt (once a month, several times a month, once a week, almost daily, never)
  - Milk (once a month, several times a month, once a week, almost daily, never)
  - Cheese (once a month, several times a month, once a week, almost daily, never)
  - Eggs (once a month, several times a month, once a week, almost daily, never)
  - Butter (once a month, several times a month, once a week, almost daily, never)
  - Juice (once a month, several times a month, once a week, almost daily, never)
  - Soft drinks (once a month, several times a month, once a week, almost daily, never)
  - Water (once a month, several times a month, once a week, almost daily, never)
  - Lemonades (once a month, several times a month, once a week, almost daily, never)
  - Coffee (once a month, several times a month, once a week, almost daily, never)
  - Tea (once a month, several times a month, once a week, almost daily, never)
  - Nonalcoholic beer (once a month, several times a month, once a week, almost daily, never)
- Wie häufig waren Sie während dieser Schwangerschaft bei einem Arzt? (How often have you consulted a doctor during pregnancy?)
- Haben oder hatten Sie jemals eine der folgenden von einem Arzt diagnostizierten Erkrankungen? (Do you have a history of somatic or psychiatric diseases?)
  - Yes: depression, ADHD, anorexia, bulimia, panic attack; allergy, asthma, atopic dermatitis, Crohn’s disease, Colitis ulcerosa, psoriasis, psoriasis arthritis, rheumatic arthritis, other autoimmune diseases, diabetes, liver- or kidney diseases, thyroid diseases, cancer, thrombosis, arrythmia, heart attack, heart failure, hypertension, pyelonephritis, metabolic diseases, migraine, multiple sclerosis, peripheral nerve paralysis, epilepsy, meningitis, encephalitis
  - No
- Ist das Ihr erstes Kind? (Is this your first child?)
- In welcher SSW haben Sie entbunden? (Which week of gestation did you deliver your baby?)
- Stillen Sie?/Haben Sie vor, zu stillen? (Are you breastfeeding or intend to do so?)
  - Yes
  - No
