# Supplementary material for: Determinants of maternal health four weeks after delivery: cross-sectional findings from the KUNO-kids health study
Source: BMC Public Health. 2021 Sep 15;21:1676. doi: 10.1186/s12889-021-11667-y (PMC8442319; doi:10.1186/s12889-021-11667-y)
Supplement: Supplementary file 2 — Additional file 2. Characteristics of the study population compared to the initial sample (mothers who participated only in the baseline interview) [file 12889_2021_11667_MOESM2_ESM.docx]

**Additional file 2:**

**Supplementary table:** Characteristics of the study population compared to the initial sample (mothers who participated only in the baseline interview)

|  | | Mothers (n=1428): study population | | | Mothers (n=2606): initial sample | | |
| --- | --- | --- | --- | --- | --- | --- | --- |
| Characteristic | | N | mean value / absolute number | [Minimum; Maximum] or % | N | mean value / absolute number | [Minimum; Maximum] or % |
| *Socioeconomic factors* | | | | | | | |
| Age (years) | | 1414 | 34.45 | 19;49 | 2575 | 34.27 | 19;50 |
| Single mother | | 1404 |  |  | 2558 |  |  |
|  | yes |  | 25 | 1.8% |  | 74 | 2.9% |
|  | no |  | 1379 | 98.2% |  | 2484 | 97.1% |
| Health insurance | | 1402 |  |  | 2554 |  |  |
|  | statutory |  | 1170 | 83.5% |  | 2176 | 85.2% |
|  | private |  | 228 | 16.3% |  | 370 | 14.5% |
|  | other |  | 4 | 0.3% |  | 8 | 0.3% |
| Educational status | | 1400 |  |  | 2550 |  |  |
|  | None |  | 2 | 0.1% |  | 22 | 0.9% |
|  | Low^[[1]](#footnote-1)^ |  | 103 | 7.4% |  | 261 | 10.2% |
|  | Medium^[[2]](#footnote-2)^ |  | 449 | 32.1% |  | 817 | 32.0% |
|  | High^[[3]](#footnote-3)^ |  | 841 | 60.1% |  | 1434 | 56.2% |
|  | Other |  | 5 | 0.4% |  | 16 | 0.6% |
| Employment before maternity leave | | 1402 |  |  | 2555 |  |  |
|  | Yes |  | 1265 | 90.2% |  | 2235 | 87.5% |
|  | No |  | 137 | 9.8% |  | 320 | 12.5% |
| Occupational group | | 1259 |  |  | 2226 |  |  |
|  | Employee |  | 1001 | 79.5% |  | 1779 | 79.9% |
|  | Worker |  | 19 | 1.5% |  | 36 | 1.6% |
|  | in training |  | 9 | 0.7% |  | 30 | 1.3% |
|  | Self-employed |  | 42 | 3.3% |  | 75 | 3.4% |
|  | civil servant |  | 165 | 13.1% |  | 259 | 11.6% |
|  | Graduate in liberal profession |  | 15 | 1.2% |  | 33 | 1,5% |
|  | Other |  | 8 | 0.7% |  | 14 | 0.6% |
| Migration background | | 1428 |  |  | 2562 |  |  |
|  | None |  | 1272 | 90.4% |  | 2171 | 84.7% |
|  | Yes |  | 135 | 9.6% |  | 391 | 15.3% |
| *Lifestyle factors* | | | | | | | |
| BMI before pregnancy | | 1412 |  |  | 2568 |  |  |
|  | underweight |  | 31 | 2.2% |  | 68 | 2.6% |
|  | normal |  | 857 | 60.7% |  | 1556 | 60.6% |
|  | overweight |  | 351 | 24.9% |  | 619 | 24.1% |
|  | obese |  | 173 | 12.3% |  | 325 | 12.7% |
| Diet |  | 1401 |  |  | 2555 |  |  |
|  | Healthy |  | 663 | 47.3% |  | 1186 | 46.4% |
|  | Rather unhealthy |  | 738 | 52.7% |  | 1369 | 53.6% |
| Physical activity | | 1414 |  |  | 2580 |  |  |
|  | None or rather seldom |  | 636 | 45.0% |  | 1236 | 47.9% |
|  | Regularly |  | 778 | 55.0% |  | 1344 | 52.1% |
| *Child and birth related factors* | | | | | | | |
| Birth mode | | 1428 |  |  | 2606 |  |  |
|  | C-section |  | 396 | 27.7% |  | 737 | 28.3% |
|  | Other |  | 1032 | 72.3% |  | 1868 | 71.7% |
| First child | | 1419 |  |  | 2589 |  |  |
|  | Yes |  | 821 | 57.9% |  | 1451 | 56.0% |
|  | No |  | 598 | 42.1% |  | 1138 | 44.0% |
| Preterm delivery | | 1420 |  |  | 2579 |  |  |
|  | Yes |  | 85 | 6.0% |  | 172 | 6.7% |
|  | No |  | 1335 | 94% |  | 2407 | 93.3% |
| Season of birth | | 1428 |  |  | 2606 |  |  |
|  | Winter |  | 696 | 48.7% |  | 1232 | 47.3% |
|  | Summer |  | 732 | 51.3% |  | 1374 | 52.7% |

1. Low: Haupt-/Volksschulabschluss in Germany [↑](#footnote-ref-1)
2. Medium: Realschulabschluss, Polytechnische Oberschule [↑](#footnote-ref-2)
3. High: Fachhochschulreife, Abitur [↑](#footnote-ref-3)
